# Clinical and functional characterization of CXCR1/CXCR2 biology in the relapse and radiotherapy resistance of primary PTEN-deficient prostate carcinoma

**DOI:** 10.1093/narcan/zcaa012

**Published:** 2020-07-03

**Authors:** Chris W D Armstrong, Jonathan A Coulter, Chee Wee Ong, Pamela J Maxwell, Steven Walker, Karl T Butterworth, Oksana Lyubomska, Silvia Berlingeri, Rebecca Gallagher, Joe M O’Sullivan, Suneil Jain, Ian G Mills, Kevin M Prise, Robert G Bristow, Melissa J LaBonte, David J J Waugh

**Affiliations:** Movember FASTMAN Centre of Excellence, Patrick G Johnston Centre for Cancer Research, School of Medicine, Dentistry and Biomedical Sciences, Queen’s University Belfast, Belfast BT9 7AE, UK; School of Pharmacy, Queen’s University Belfast, Belfast, BT9 7AE, UK; Laboratory of Cancer Epigenome, Division of Medical Science, National Cancer Centre, Singapore, 169610; Movember FASTMAN Centre of Excellence, Patrick G Johnston Centre for Cancer Research, School of Medicine, Dentistry and Biomedical Sciences, Queen’s University Belfast, Belfast BT9 7AE, UK; Movember FASTMAN Centre of Excellence, Patrick G Johnston Centre for Cancer Research, School of Medicine, Dentistry and Biomedical Sciences, Queen’s University Belfast, Belfast BT9 7AE, UK; Almac Diagnostics, Craigavon, BT63 5QD, UK; Movember FASTMAN Centre of Excellence, Patrick G Johnston Centre for Cancer Research, School of Medicine, Dentistry and Biomedical Sciences, Queen’s University Belfast, Belfast BT9 7AE, UK; Movember FASTMAN Centre of Excellence, Patrick G Johnston Centre for Cancer Research, School of Medicine, Dentistry and Biomedical Sciences, Queen’s University Belfast, Belfast BT9 7AE, UK; Movember FASTMAN Centre of Excellence, Patrick G Johnston Centre for Cancer Research, School of Medicine, Dentistry and Biomedical Sciences, Queen’s University Belfast, Belfast BT9 7AE, UK; Movember FASTMAN Centre of Excellence, Patrick G Johnston Centre for Cancer Research, School of Medicine, Dentistry and Biomedical Sciences, Queen’s University Belfast, Belfast BT9 7AE, UK; Movember FASTMAN Centre of Excellence, Patrick G Johnston Centre for Cancer Research, School of Medicine, Dentistry and Biomedical Sciences, Queen’s University Belfast, Belfast BT9 7AE, UK; Movember FASTMAN Centre of Excellence, Patrick G Johnston Centre for Cancer Research, School of Medicine, Dentistry and Biomedical Sciences, Queen’s University Belfast, Belfast BT9 7AE, UK; Movember FASTMAN Centre of Excellence, Patrick G Johnston Centre for Cancer Research, School of Medicine, Dentistry and Biomedical Sciences, Queen’s University Belfast, Belfast BT9 7AE, UK; Nuffield Department of Surgical Sciences, University of Oxford, OX3 9DU, UK; Movember FASTMAN Centre of Excellence, Patrick G Johnston Centre for Cancer Research, School of Medicine, Dentistry and Biomedical Sciences, Queen’s University Belfast, Belfast BT9 7AE, UK; Movember FASTMAN Centre of Excellence, Manchester CRUK Institute, Manchester, SK10 4TG, UK; Christie NHS Foundation Trust and Division of Cancer Sciences, Faculty of Biology, Medicine and Health, University of Manchester, Manchester, M20 4BX, UK; Movember FASTMAN Centre of Excellence, Patrick G Johnston Centre for Cancer Research, School of Medicine, Dentistry and Biomedical Sciences, Queen’s University Belfast, Belfast BT9 7AE, UK; Movember FASTMAN Centre of Excellence, Patrick G Johnston Centre for Cancer Research, School of Medicine, Dentistry and Biomedical Sciences, Queen’s University Belfast, Belfast BT9 7AE, UK; School of Biomedical Sciences, Faculty of Health, Queensland University of Technology, Brisbane, Queensland, QLD 4000, Australia

## Abstract

Functional impairment of the tumour suppressor *PTEN* is common in primary prostate cancer and has been linked to relapse post-radiotherapy (post-RT). Pre-clinical modelling supports elevated CXC chemokine signalling as a critical mediator of *PTEN*-depleted disease progression and therapeutic resistance. We assessed the correlation of *PTEN* deficiency with CXC chemokine signalling and its association with clinical outcomes. Gene expression analysis characterized a *PTEN*^LOW^/CXCR1^HIGH^/CXCR2^HIGH^ cluster of tumours that associates with earlier time to biochemical recurrence [hazard ratio (HR) 5.87 and 2.65, respectively] and development of systemic metastasis (HR 3.51). *In vitro*, CXCL signalling was further amplified following exposure of *PTEN*-deficient prostate cancer cell lines to ionizing radiation (IR). Inhibition of CXCR1/2 signalling in *PTEN-*depleted cell-based models increased IR sensitivity. *In vivo*, administration of a CXCR1/2-targeted pepducin (x1/2pal-i3), or CXCR2-specific antagonist (AZD5069), in combination with IR to *PTEN*-deficient xenografts attenuated tumour growth and progression compared to control or IR alone. Post-mortem analysis confirmed that x1/2pal-i3 administration attenuated IR-induced CXCL signalling and anti-apoptotic protein expression. Interventions targeting CXC chemokine signalling may provide an effective strategy to combine with RT in locally advanced prostate cancer patients with known presence of *PTEN*-deficient foci.

## INTRODUCTION

External beam radiotherapy (RT) constitutes a principal treatment modality for organ-confined prostate cancer (CaP) (1). Although the majority of tumours respond favourably, over one-third of patients will experience relapse post-RT, which has been attributed to intrinsic radioresistance of tumour cells, release and signalling of stroma-derived survival factors or presence of occult micro-metastases at the time of diagnosis (2–4). The cellular response of tumour cells to DNA damage treatment can be related to an underlying genetic background and can itself markedly alter gene expression to effect differential phenotypic behaviour (5,6). Crucially, RT is no longer restricted to use in the treatment of local disease but is becoming a viable therapeutic option for advanced disease, with clinical trials currently evaluating the potential use of stereotactic RT to treat oligometastatic CaP (7–9). The extended deployment of radiation as an intervention across the continuum of the clinical landscape accentuates the requirement to optimize this treatment modality.

Improving the effectiveness of RT has typically followed two distinct pathways: (i) altering standard treatment protocols with the intent to boost the overall radiation dosage delivered to the tumour, including the recent introduction of hypofractionated dose scheduling (10–12), and (ii) the use of genetic markers to stratify patients and/or progress combination-targeted therapy to attenuate associated survival mechanisms adopted by cells as a mechanism of resistance to radiation-induced cell death. Zafarana *et al.* originally reported allelic loss of the tumour suppressor *PTEN* and allelic gain of *c-MYC* as prognostic factors for relapse following RT (13). Identifying the critical signalling pathways that underpin and confer *PTEN*-mediated resistance is essential to defining actionable combinatorial drug–RT treatment approaches that may be employed to improve RT response in future patients (14,15).

*PTEN*, a potent negative regulator of the PI3K–Akt signalling axis, is deleted or mutated in ∼30% of men with localized CaP and in over 60% of patients exhibiting metastatic progression (16,17). Moreover, impairment of PTEN function is associated with clinico-pathological features of aggressive and treatment-resistant prostate carcinoma (13,18,19). Our initial studies confirmed the increased expression of CXCL8 and its two receptors CXCR1 and CXCR2 in the tumour epithelium of human CaP (20). Our subsequent studies in human CaP cell lines and genetically engineered mouse models associated the elevated expression of this chemokine signalling pathway with *PTEN* loss (21). Intrinsic CXCL8 signalling underpins prostate cancer cell survival through the activation of AR, HIF-1 and NF-κB transcription factors, and increases expression of anti-apoptotic proteins, including members of the Bcl family (22). In addition to underpinning resistance to AR-targeted therapy, induction of CXCL8 signalling modulates the sensitivity of prostate cancer cells to several novel molecular targeted therapies and chemotherapeutic agents, including oxaliplatin that induces DNA double-strand breaks in CaP cells (23). Thus, we adopted the hypothesis that exposure to ionizing radiation (IR) would similarly induce chemokine signalling and that this would have a profound impact in modulating the sensitivity of *PTEN*-deficient tumours to radiation.

The objective of this comprehensive study, employing clinical datasets and established *in vitro* and *in vivo* models, was to characterize whether stress-induced potentiation of CXCR1/2 signalling may underpin the adverse response of *PTEN*-deficient prostate cancer to radiation and to potentially explain biological mechanisms related to increased clinical relapse reported in *PTEN*-deficient tumours.

## MATERIALS AND METHODS

### Cell culture

Authenticated DU145, LNCaP, C4-2, C4-2B, PC3 and 22Rv1 CaP cells were obtained from ATCC. DU145 cells were manipulated as previously described to generate isogenic PTEN-expressing NT01 cells and PTEN-deficient sh11.02 cells (21). PC3 cells were manipulated as previously described so that PTEN expression can be reconstituted following exposure to tetracycline (21). PTEN-depleted 22Rv1 cells were generated following lentiviral transfection of HuSh-29 pre-designed PTEN shRNA pGFP-V-RS constructs (Origene, Rockville, MD, USA), and selected under puromycin selection pressure at a final concentration of 0.5 μg/ml. Cell line authenticity was confirmed by STR genotyping (July 2019) and mycoplasma testing was performed every 4–6 weeks (MycoAlert, Lonza).

### Radiation treatments

The X-RAD 225 series irradiator was used to carry out all *in vitro* experiments described. This cabinet contains a high-voltage generator with a maximum output voltage of 225 kV. The X-ray tube is a metal ceramic fixed anode that is water cooled and has a maximum potential of 225 kV. The operating conditions of the machine were 225 kV and 13.3 mA. All experiments were performed using the 50 cm shelf position, which resulted in an output of 0.588 Gy/min. Cell treatments were therefore calculated using the following formula:

$$\begin{equation*}{\rm time} = {\rm required}\,{\rm dose}/0.588.\end{equation*}$$

Radiation treatments were also administered to NT01, sh11.02 and PC3 xenograft models using the X-RAD 225. Lead shielding was used to minimize off-target effects. For the C4-2 xenograft model, CT-guided RT was delivered using a Small Animal Radiation Research Platform (XStrahl, Camberley, UK).

### ELISA

Cells were plated into six-well plates at a density of 5 × 10^5^ cells per well and allowed to adhere overnight. After 24 h, cells were irradiated and media samples collected at various time points. Cell counts were performed at each time point. CXCL8 ELISA experiments were performed using DuoSet^®^ ELISA Development Kits (R&D Systems) according to manufacturer’s instructions. CXCL8 secretion was normalized to cell count to correct for differences in confluency.

### Immunoblotting

Whole cell lysates were prepared, resolved and blotted as previously described (21). Membranes were probed with primary antibodies at 4°C overnight. Primary antibody information can be found in Supplementary Table S1. Following three TBST washes, membranes were incubated with the appropriate horseradish peroxidase-labelled secondary antibody (1:5000 dilution; GE Healthcare UK Ltd, UK). Protein bands were detected using enhanced chemiluminescence (Luminata Crescendo, Merck Millipore). Membranes were re-probed with β-Actin antibody to ensure equal loading.

### Quantitative real-time PCR

Total RNA was collected and isolated as previously described (21). Quantitative real-time polymerase chain reaction (qRT-PCR) was performed using pre-validated RealTime ready custom assays for *CXCR1*, *CXCR2*, *CXCL8* and *BCL2* used in combination with FastStart TaqMan^®^ Probe Master solutions (Roche Diagnostics, Sussex, UK). Individual sample mRNA levels were analysed in triplicate in a 96-well plate using an LC480 light cycler instrument (Roche Diagnostics). Gene expression levels were normalized against 18S.

### siRNA

siRNA transfections for CXCR1 and CXCR2 oligonucleotides (Dharmacon, Lafayette, CO, USA) were carried out using Lipofectamine^®^ RNAiMAX Transfection Reagent (Life Technologies, Paisley, UK) when cells reached 60–70% confluence. Briefly, for a p90 Petri dish, 10 μl RNAiMAX was combined with 25 nM pooled CXCR1 and CXCR2 oligonucleotides (12.5 nM CXCR1/12.5 nM CXCR2) and added in a drop-wise fashion to 2 ml Opti-MEM. Transfection complexes were then incubated at room temperature for 20 min, after which the siRNA complexes were added to 8 ml of complete medium. Cells were then maintained at 37°C for 48 h. Non-targeting sequences were used at the same concentration (25 nM) as the total combined CXCR1 and CXCR2 siRNA sequences.

### Flow cytometry

Cells were seeded at a density of 5 × 10^4^ per well in a six-well plate and left to adhere overnight. Cells were then transfected with the appropriate control or siRNA oligonucleotides and returned to the incubator. After 72 h, all groups received either a 3 Gy radiation dose or sham irradiation. Cells were analysed 72 h post-radiation treatment. Whole culture medium was collected and pooled with the trypsinized cells, and then centrifuged at 1500 rpm. Cell pellets were resuspended in 100 μl of 1× binding buffer. Annexin V (Life Technologies, Paisley, UK) antibody (5 μl) was added to each sample along with 5 μl of propidium iodide (PI) stain (50 μg/ml). Samples were then incubated in the dark, at room temperature for 15 min. After incubation, 320 μl of 1× binding buffer was added to each sample before analysis on the EPICS XL flow cytometer (Beckman Coulter, Buckinghamshire, UK).

### Clonogenic assays

Reverse clonogenic assays were performed as follows. In CXCR1/2-targeting experiments, cells were transfected, irradiated 48 h post-transfection and reseeded to assess colony-forming ability. In PC3-PTEN cells, transfections were performed 24 h prior to *PTEN* reconstitution using tetracycline (1 μg/ml). Surviving fractions were calculated relative to non-irradiated cells and fitted using a linear quadratic function [*S* = exp(−*αD* − *βD*^2^)] using least-squares regression (Prism 7.0; GraphPad Software, San Diego, CA, USA). Area under the curve representing the mean inactivation dose (MID) was obtained and dose enhancement factor (DEF) calculated by dividing the MID of the CXCR1/2-depleted cells by that of non-targeting siRNA-treated cells.

### PC3 and DU145 xenograft tumour growth delay models

CXCR1/2-blocking pepducins (CXCR1/2 x1/2pal-i3 pepducin—sequence pal-RTLFKAHMGQKHR-NH_2_; non-targeting x1/2pal-con—sequence pal-TRFLAKMHQGHKR-NH_2_) that target the highly conserved third intracellular loop were used to block chemokine receptor-mediated signalling *in vivo*. Pepducin targeting and efficacy were confirmed *in vitro* prior to *in vivo* use. PC3, NT01 or sh11.02 cells [2 × 10^6^ in phosphate-buffered saline (PBS)] were implanted by intradermal injection on the rear dorsum of BALB/c SCID mice (Envigo). Animals with palpable tumours (100 mm^3^) were randomized to treatment groups (*N* = 7 per group): no treatment, x1/2pal-con, x1/2pal-i3, 3 Gy, x1/2pal-con + 3 Gy and x1/2pal-i3 + 3 Gy. Pepducins, reconstituted in PBS, were administered by intratumoural injection (2 mg/kg) on days 1, 2, 3, 4 and 5. Radiation was administered on day 3. Animals were restrained in a Perspex jig and protected from non-target radiation damage using lead shielding. Radiation treatments were delivered as two parallel-opposed fields using a Precision X-RAD 225. Due to the increased radiation sensitivity of DU145 cells, radiation dose was reduced to 2 Gy. Tumour dimensions were measured using callipers and tumour volumes calculated using the following formula: (width^2^ × length)/2. Tumour and weight measurements were performed every Monday, Wednesday and Friday for the duration of the study. Animals were culled when the tumour volume quadrupled (400 mm^3^). Additional mice (*N* = 4 per group) were culled on study day 5 to enable collection of tumours for pharmacokinetic analysis. Tumours were cut into two halves and stored in either formalin or liquid nitrogen.

### C4-2 xenograft model

SCID male mice (7 weeks old) were obtained from Envigo. C4-2 cells were injected subcutaneously, 1 × 10^7^ cells in 100 μl PBS:Matrigel (50:50) in the right flank. Once palpable tumours formed, tumour volume was measured by digital callipers, as described earlier. When tumours reached 100 mm^3^, mice were randomized to treatment groups (*N* = 8 per group): vehicle control, AZD5069, 3 Gy or AZD5069 + 3 Gy. AZD5069 was prepared in 1× PBS/0.1% Tween 20 and administered by oral gavage at 2 mg/kg daily. Radiation was administered on day 3, as described earlier. Tumour and weight measurements were performed thrice weekly for the duration of the study. Animals were culled when the tumour volume reached maximum (1000 mm^3^). Additional mice (*N* = 4 per group) were culled on study day 5 to enable collection of tumours for pharmacokinetic analysis. Tumours were cut into two halves and stored in either formalin or liquid nitrogen.

### 53BP1 foci immunofluorescence

Cells (1 × 10^4^) were seeded onto four-well chamber slides (Fisher Scientific, Leicestershire, UK) and left overnight to adhere. For *PTEN*-depleted damage repair studies, cells were irradiated with 1 Gy (to prevent damage saturation) and analysed at indicated time points ranging for 0.5 to 24 h post-radiation to observe repair kinetics. For CXCR1/CXCR2 knockdown experiments, transfections were performed 72 h prior to irradiation. Cells were fixed 4 h post-radiation treatment with 50% methanol:50% acetone, and permeabilized in 0.5% Triton X-100 (Sigma, Dorset, UK). After an overnight incubation in blocking buffer (PBS, 0.1% Triton X-100, 5% FCS, 0.2% milk), cells were incubated with rabbit anti-53BP1 antibody (Abcam, Cambridge, UK) at 1:2000 concentration and incubated with secondary anti-rabbit Alexa Fluor 488 (Life Technologies, Paisley, UK). Nuclei were counterstained with 4,6-diamidino-2-phenylindole (0.1 mg/ml). Foci were counted using a fluorescence microscope (Zeiss Axiovert 200M, UK); typically, 100 cells were counted per treatment condition.

### Immunohistochemistry

Sections were cut from tumour tissue blocks for haematoxylin and eosin (H&E) staining and immunohistochemistry (IHC). The initial section was used for H&E staining to assess histology and appropriate tumour content for subsequent IHC localization and analysis. Sections for IHC were cut at 4 μM on a rotary microtome, dried at 37°C overnight and used for IHC tests, performed on an automated immunostainer (Leica BOND-MAX™). Validated and optimized protocols, used in local diagnostics, were selected for each biomarker with inclusion of carefully selected control tissues during antibody application. Antigen binding sites were detected with a polymer-based detection system (Bond cat. no. DS 9800). All sections were visualized with 3,3′-diaminobenzidine, counterstained in haematoxylin and mounted in DPX. Biomarker conditions were as follows: Ki-67 (clone MM1 DO-7 mouse monoclonal antibody, Leica) was used at a 1:200 dilution with epitope retrieval solution 2 pre-treatment for 30 min. Bcl-2 (clone 3.1 mouse monoclonal antibody, Leica) was used at a 1:100 dilution with epitope retrieval solution 2 pre-treatment for 20 min.

### Statistical analysis

Unless otherwise stated, experiments were performed in triplicate and results expressed as mean ± standard error (SE). Data were analysed using a two-tailed unpaired *t*-test or two-way ANOVA (clonogenic survival curves) with a *P*-value of <0.05 considered to be statistically significant.

## RESULTS

### Correlation of *PTEN* and CXC chemokine signalling to clinical parameters of progression

**Figure 1.**
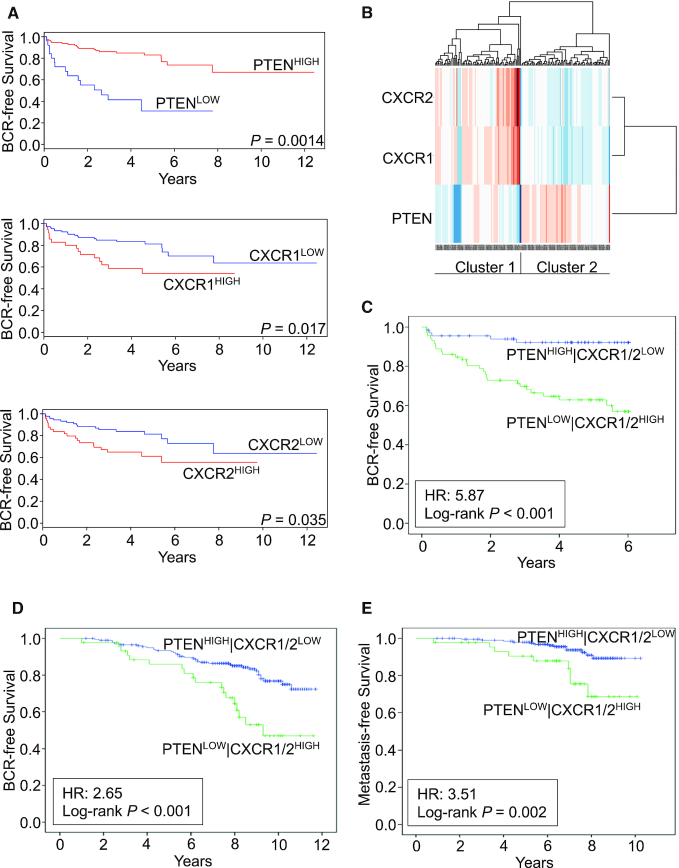
*PTEN^LOW^*, *CXCR1^HIGH^* and *CXCR2^HIGH^* tumours are associated with poor prognosis. (**A**) Kaplan–Meier curves examining the relevance of *PTEN*, *CXCR1* and *CXCR2* expression independently in the Taylor (MSKCC) dataset for BCR. High and low expressions of each gene were determined based on differences from the median threshold (*PTEN*: 8.63; *CXCR1*: 6.27; *CXCR2*: 6.59). Sample sizes were as follows: *PTEN^HIGH^* (*N* = 114); *PTEN^LOW^* (*N* = 26); *CXCR1^HIGH^* (*N* = 35); *CXCR1^LOW^* (*N* = 105); *CXCR2^HIGH^* (*N* = 50); *CXCR2^LOW^* (*N* = 90). (**B**) Cluster analysis of *PTEN*, *CXCR1* and *CXCR2* in the MSKCC dataset. (**C**) Kaplan–Meier survival curves in 140 patients from the MSKCC dataset in relation to sample clustering by three genes (*PTEN*, *CXCR1* and *CXCR2*). Sample sizes were as follows: *PTEN^HIGH^|CXCR1/2^LOW^* (*N* = 66); *PTEN^LOW^|CXCR1/2^HIGH^* (*N* = 74). (**D**, **E**) Analysis of the FASTMAN retrospective RT patient sample cohort (*N* = 248) showing the impact of previously defined clusters on time to recurrence and time to metastasis, respectively. Sample sizes were as follows: *PTEN^HIGH^|CXCR1/2^LOW^* (*N* = 203); *PTEN^LOW^|CXCR1/2^HIGH^* (*N* = 45). Data information: significant differences were determined by the log-rank test. Abbreviations: BCR, biochemical recurrence; HR, hazard ratio.

*PTEN* deficiency increases *CXCL8*, *CXCR1* and *CXCR2* gene expression in human CaP cell lines *in vitro* and that of the orthologous pathway in the prostate epithelium of *PTEN*^+/−^ mice (21). We conducted *in silico* analysis of publicly available CaP datasets to confirm this association in human prostate cancer and its association with clinical outcomes. Our initial analysis was conducted using the MSKCC radical prostatectomy cohort, focusing on the 140 patients with relevant clinical follow-up data (24). This dataset derived from radically resected tumours demonstrated that *PTEN^LOW^* (*P* = 0.0014), CXCR1^HIGH^ (*P* = 0.017) and CXCR2^HIGH^ (*P* = 0.035) expression each independently correlated with accelerated biochemical recurrence (BCR; Figure 1A). We observed that the *PTEN*^LOW^ and CXCR1/2^HIGH^ tumours formed a distinct cluster or subgroup constituting 74 (52.9%) of the 140 tumours represented in this resection cohort (Figure 1B). Kaplan–Meier analysis revealed that the PTEN^LOW^/CXCR1/2^HIGH^ cluster was associated with a highly significant reduction in time to BCR [*P* < 0.001; hazard ratio (HR) 5.87; Figure 1C]. Similar analysis was performed on the TCGA dataset that confirmed the prognostic effect of PTEN loss and the combined PTEN^LOW^/CXCR1/2^HIGH^ signature (Supplementary Figure S5).

Further analysis was performed on a third dataset to determine the direct relevance of this gene cluster with respect to RT response. Analysis was conducted on a transcriptomic profile derived from the FASTMAN retrospective RT patient sample cohort (25). This cohort of 248 diagnostic biopsy samples has a median follow-up data of >100 months. Kaplan–Meier analysis confirmed that *PTEN*^LOW^ and CXCR1/2^HIGH^ tumours were associated with a significantly reduced time to BCR (Figure 1D; *P* < 0.001; HR 2.65) and importantly with the development of metastasis (Figure 1E; *P* = 0.002; HR 3.51) after RT treatment. Taken together, these results establish the clinical relevance of the PTEN^LOW^/CXCR1/2^HIGH^ cluster in two distinct cohorts and, furthermore, reveal the downstream significance of this biology in locally advanced prostate cancer to adverse outcomes after both surgery and RT interventions.

### Expression of *PTEN* modulates radiation-induced CXCL chemokine signalling

**Figure 2.**
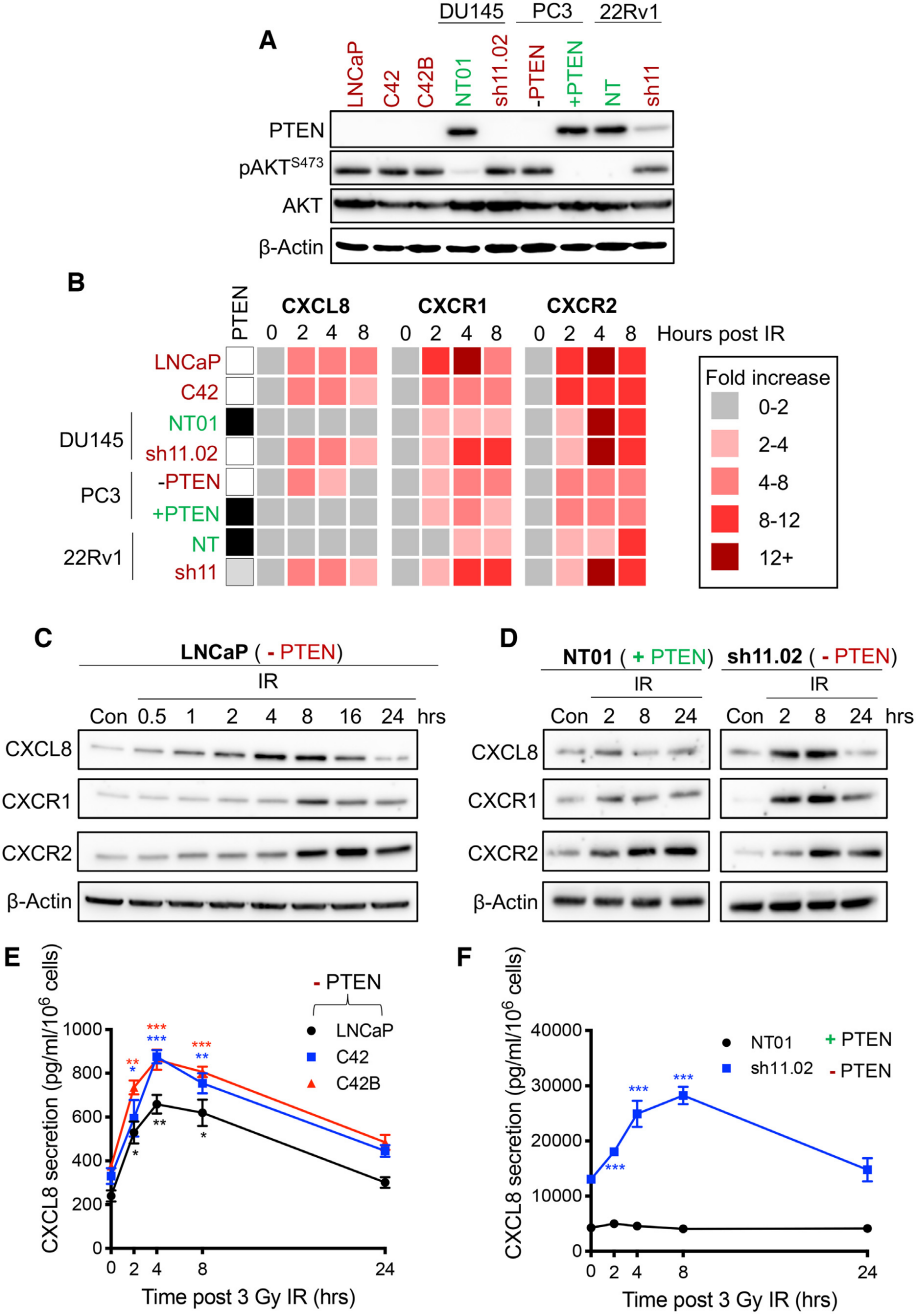
IR induces CXC chemokine expression and secretion in *PTEN*-deficient prostate cancer models. (**A**) Immunoblots showing expression of PTEN, pAKT (Ser473) and AKT in a panel of prostate cancer cell models. PTEN expression was depleted in DU145 and 22Rv1 cells using a lentiviral-based protocol. PTEN expression was reconstituted in PC3 cells under the control of a tetracycline-inducible promoter. Equal loading was confirmed by re-probing blots for β-Actin. (**B**) Heat map showing fold increase of *CXCL8*, *CXCR1* and *CXCR2* gene expression from 0 to 8 h following exposure to 3 Gy IR. PTEN expression of each cell line is indicated in the left panel (white: PTEN-null; black: PTEN-expressing; grey: partial PTEN depletion). (**C**, **D**) Immunoblots showing expression of CXCL8, CXCR1 and CXCR2 at 0–24 h following treatment LNCaP, NT01 and sh11.02 cells with 3 Gy IR. Equal loading was confirmed by re-probing blots for β-Actin. (**E**) Graph showing CXCL8 secretion from LNCaP, C4-2 and C4-2B cells from 0 to 24 h following treatment with 3 Gy IR. (**F**) Graph showing CXCL8 secretion from NT01 and sh11.02 cells from 0 to 24 h following treatment with 3 Gy IR. Data information: all data presented are a representation of *N* = 3 independent experiments. (E, F) Statistical analysis is a comparison of each time point to the 0 h control within the same cell line (*P < 0.05; **P < 0.01; ***P < 0.001).

RT is a major treatment modality for locally advanced and increasingly for oligometastatic prostate cancer. Experiments were therefore performed across a range of CaP cell models representative of different androgen sensitivity, different metastatic potential and exhibiting differential expression of PTEN: we used *PTEN*-null LNCaP and LNCaP-derived C4-2 and C4-2B cells and isogenic PTEN-expressing and -null cell lines (DU145, PC3 and 22Rv1) (21). Their PTEN expression and resulting downstream PI3K–Akt signalling axis activity were confirmed by immunoblot analysis for PTEN and phosphorylation status of AKT^S473^ (Figure 2A).

The role of radiation in modulating CXC chemokine signalling was investigated using PTEN-expressing and -null CaP cells, exposed to clinically relevant doses of radiation (2–3 Gy). qRT-PCR analysis was used to quantify alterations in *CXCL8*, *CXCR1* and *CXCR2* gene expression (Figure 2B). While *CXCL8* expression remained unchanged in cell lines that retained sufficient levels of *PTEN* (DU145-NT01, PC3-PTEN and 22Rv1-NT), PTEN-deficient cells demonstrated an increase in *CXCL8* mRNA 2 h post-radiation that was sustained out to 8 h. However, treatment with IR induced the expression of chemokine receptors *CXCR1* and *CXCR2* in tumour cells independent of *PTEN* status (Figure 2B).

Similar experiments were performed to assess the impact of IR on CXC chemokine protein expression using LNCaP and DU145-NT01 and sh11.02 cells. Treatment with 3 Gy increased CXCL8 expression in *PTEN*-null LNCaP and *PTEN*-depleted sh11.02 cells, but did not modulate expression in *PTEN*-expressing NT01 cells. Confirming our qRT-PCR analysis, IR-induced expression of CXCR1 and CXCR2 at the protein level was independent of intrinsic PTEN status (Figure 2C and D).

We further assessed the effect of IR on CXCL8 secretion. Exposure to IR induced a 3–4-fold increase in the secretion of this chemokine from *PTEN*-null LNCaP, C4-2 and C4-2B cells. This was evident 2 h post-IR exposure, with levels returning to baseline within 24 h (Figure 2E). Similarly, in PTEN-expressing DU145-NT01 cells and PTEN-depleted DU145-sh11.02 cells, treatment with 3 Gy resulted in significantly increased CXCL8 secretion in sh11.02 cells, but had no effect in NT01 cells (Figure 2F). Interestingly, baseline secretion was already 3.06-fold greater (*P* < 0.0001) in the PTEN-depleted sh11.02 cells (Figure 2F). Crucially, we observed these increases in CXCL8 secretion across androgen-dependent and -independent models signifying the importance of this biology across the disease spectrum.

### Inhibition of CXCR1/2 signalling promotes *PTEN*-dependent radiosensitization

**Figure 3.**
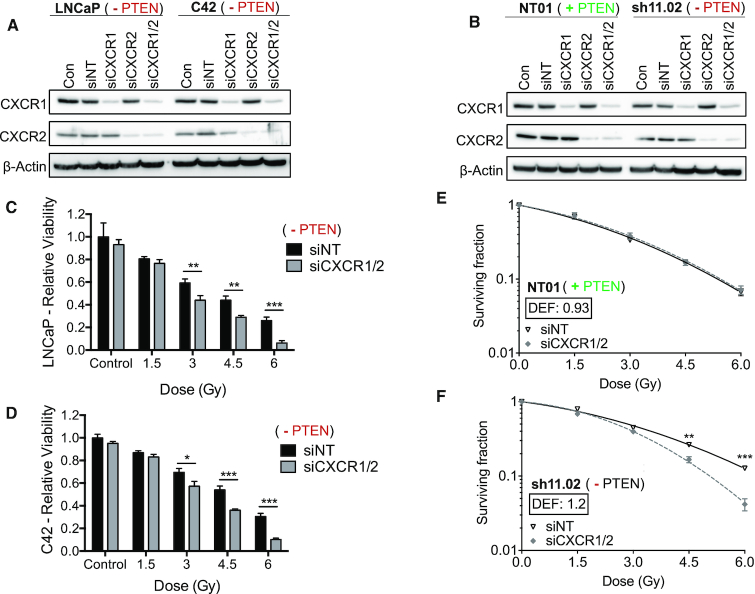
Radiosensitivity of *PTEN*-deficient prostate cancer via CXCR1/2 knockdown. (**A**, **B**) Immunoblots showing CXCR1 and CXCR2 expression following treatment with non-targeting siRNA (siNT), CXCR1 siRNA (siCXCR1), CXCR2 siRNA (siCXCR2) or a combination of both CXCR1 and CXCR2 siRNA (siCXCR1/2). Final siRNA concentration was 25 nM. Equal loading was confirmed by re-probing blots for β-Actin. (**C**, **D**) Bar charts showing relative viability of LNCaP and C4-2 cells, respectively, as determined by Alamar Blue assay. Cells were pre-treated with 25 nM non-targeting siRNA (siNT) or CXCR1 and CXCR2 siRNA (siCXCR1/2) for 48 h prior to treatment with IR (0–6 Gy dose range). Samples were analysed 7 days following IR treatment. (**E**, **F**) Clonogenic survival curves generated from NT01 and sh11.02 cells, respectively. Cells were pre-treated with 25 nM non-targeting siRNA (siNT) or CXCR1 and CXCR2 siRNA (siCXCR1/2) for 48 h prior to IR treatment. Data information: all data presented are a representation of *N* = 3 independent experiments. All survival curves are fitted to a linear quadratic model and DEF was calculated using the MID based on the area under the curve at a surviving fraction of 0.1. Statistically significant differences were determined using *t*-tests (*P < 0.05; **P < 0.01; ***P < 0.001).

CXCR1- and CXCR2-targeted siRNA was used to knock down expression of both receptors in order to assess the survival advantage afforded by hyperactive CXC chemokine signalling. Validation of these siRNAs confirmed their ability to successfully reduce expression of the respective receptors by >90% in LNCaP and C4-2 cells (Figure 3A). Knockdown of both receptors was also validated in PTEN-expressing DU145-NT01 cells and PTEN-depleted DU145-sh11.02 cells (Figure 3B), providing a range of experimental models to evaluate the impact of targeting CXCL8/CXCR signalling.

The impact of *PTEN* depletion and/or repression of CXCL8 signalling on cell proliferation was determined initially by cell growth curve analysis. Knockdown of CXCR1 and CXCR2 alone had limited impact on the cell proliferation kinetics of either the PTEN-expressing DU145-NT01 or PTEN-depleted sh11.02 cells. However, siRNA-mediated suppression of CXCR1/2 enhanced the effects of IR in both the NT01 and sh11.02 cells, increasing the observed cell doubling time by 2.2- and >3-fold, respectively (Supplementary Table S2).

Due to the inherently poor colony-forming ability of LNCaP cells, we used an Alamar Blue assay to assess the impact of CXCR1/2 knockdown upon the viability of these cells at 7 days post-radiation. Compared to cells treated with non-targeting siRNA, knockdown of both receptors significantly decreased cell viability in LNCaP and C4-2 cells once exposed to doses >3 Gy (Figure 3C and D).

Cells derived from the DU145 lineage have excellent colony-forming ability and so clonogenic survival assays were used to confirm the survival advantage associated with sustained CXCR1/2 signalling. Knockdown of these receptors in PTEN-expressing NT01 cells resulted in no significant difference in survival following exposure to IR (DEF = 0.93; Figure 3E). However, siRNA-mediated CXCR1/2 knockdown in *PTEN*-depleted sh11.02 cells increased sensitivity to IR with a calculated DEF of 1.2 (Figure 3F). Conversely, we employed a PTEN-inducible PC3 cell model, whereby the radiosensitizing effect of CXCR1/2 siRNA detected in the PTEN-null parental cell line was completely ablated following reconstitution of PTEN expression (Supplementary Figure S1).

Combined, these results confirm that a CXCR1/2-targeted therapeutic approach can drive radiosensitivity in *PTEN*-depleted prostate cancer models.

### *PTEN* depletion combined with knockdown of CXCR1/2 impairs tumour cell proliferation and promotes apoptosis

**Figure 4.**
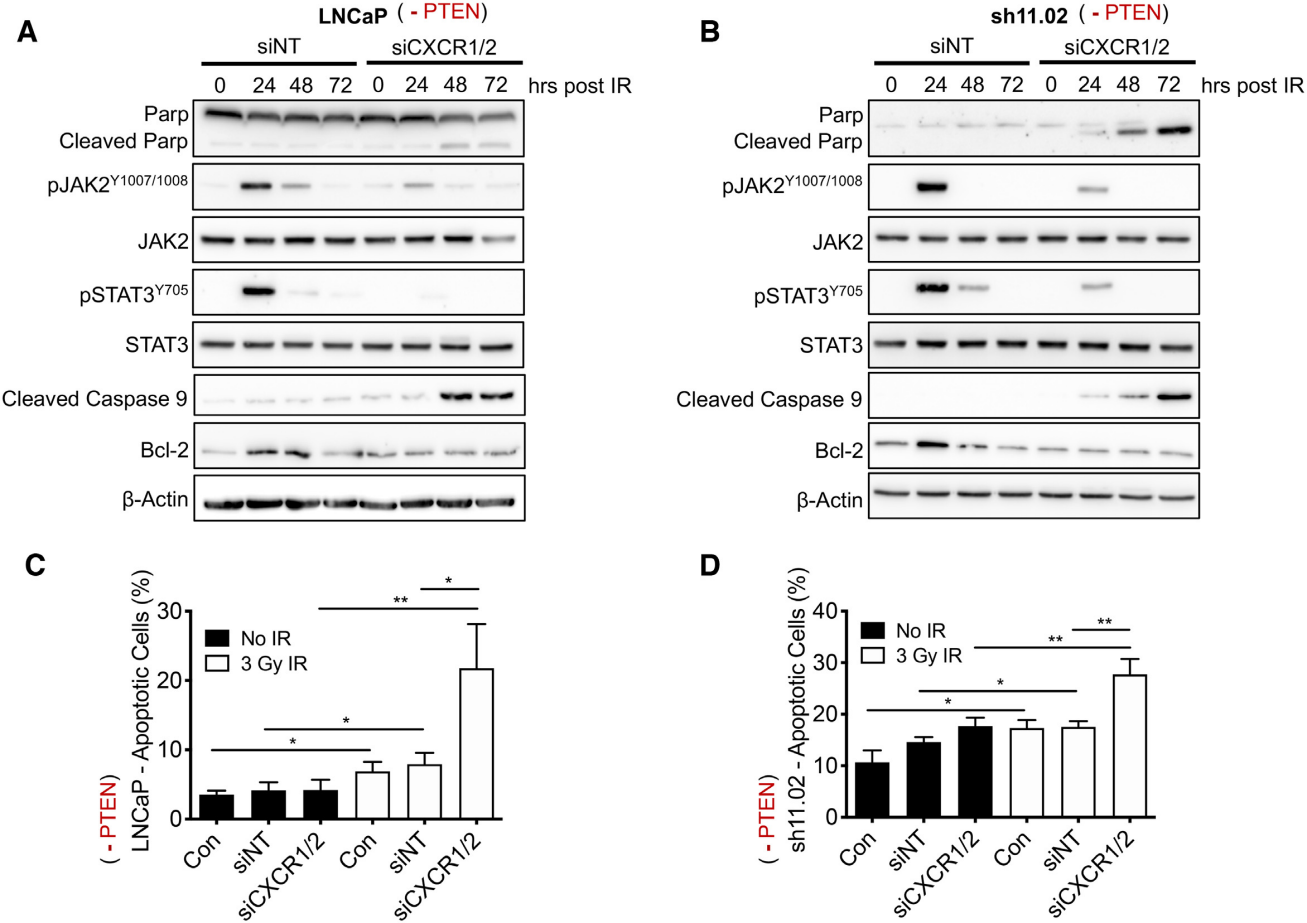
Knockdown of CXCR1 and CXCR2 enables the induction of apoptosis following treatment with radiation. (**A**, **B**) Immunoblots examining expression of an apoptotic protein panel in LNCaP and DU145-sh11.02 cells, respectively. Cells were pre-treated with 25 nM non-targeting siRNA (siNT) or CXCR1 and CXCR2 siRNA (siCXCR1/2) for 48 h prior to treatment with 3 Gy IR. Equal loading was confirmed by re-probing blots for β-Actin. (**C**, **D**) Bar charts summarizing Annexin V/PI flow cytometry analysis of LNCaP and sh11.02 cells, respectively. Cells were pre-treated with 25 nM non-targeting siRNA (siNT) or CXCR1 and CXCR2 siRNA (siCXCR1/2) for 48 h prior to treatment with 3 Gy IR. Cells were analysed 72 h following IR treatment alongside non-irradiated control cells (black bars). Data information: all data presented are a representation of *N* = 3 independent experiments. Statistically significant differences were determined using *t*-tests (*P < 0.05; **P < 0.01; ***P < 0.001).

We have previously shown that CXCR1/2-mediated signalling results in upregulation of the anti-apoptotic, pro-survival protein, Bcl-2, suggesting that CXCR1/2 knockdown may confer radiosensitivity through modulation of the apoptotic pathway (21). Immunoblotting confirmed that exposure to IR induced Bcl-2 expression in androgen-dependent LNCaP cells. We also observed increased JAK2^Y1007/1008^ and STAT3^Y705^ phosphorylation providing a potential mechanism for potentiation of anti-apoptotic pathways (26). However, knockdown of CXCR1/2 prior to IR treatment repressed the ability of LNCaP cells to induce JAK/STAT phosphorylation and subsequent Bcl-2 expression (Figure 4A). This coincided with increased detection of cleaved PARP and caspase 9, confirming the induction of apoptosis. Similar immunoblotting profiles were observed when experiments were repeated in the androgen-independent *PTEN*-deficient DU145-sh11.02 cells (Figure 4B).

Increased apoptotic signalling was validated by analysis of apoptotic fractions using Annexin V/PI staining protocols in LNCaP and DU145-sh11.02 cells. Treatment with 3 Gy IR increased the apoptotic fraction of LNCaP cells from 3.53% to 6.90% (*P* = 0.0174) and non-targeting siRNA-treated LNCaP cells from 4.16% to 7.93% (*P* = 0.0305). However, the most significant effect was observed in cells transfected with CXCR1/2 siRNA, where concurrent exposure to IR increased the apoptotic cell fraction from 4.20% to 21.76% (*P* = 0.0097; Figure 4C). Similar results were obtained in DU145-sh11.02 cells, where the addition of IR increased the apoptotic fraction of CXCR1/2 siRNA-treated cells from 17.66% to 27.70% (*P* = 0.0071; Figure 4D).

Additional experiments were performed to assess the DNA damage response following IR exposure and CXCR1/2 inhibition. Interestingly, PTEN-deficient sh11.02 cells had higher basal levels of 53BP1 foci and a slower rate of repair following exposure to 1 Gy IR compared to PTEN-expressing NT01 cells. However, attenuation of CXCR1/2 signalling had no additional effect on the DNA damage response (Supplementary Figure S2).

### *PTEN* loss and combined CXCR1/2 inhibition with radiation slows tumour growth in androgen-independent xenografts

Further experiments were conducted to evaluate the impact of CXCR1/2 inhibition upon the response of growing prostate tumours to RT. In these experiments, rather than using siRNA to deplete CXCR1/2 signalling potential, we employed CXCR1/2-targeted peptidomimetics termed ‘pepducins’ that have been shown to uncouple the receptors from activating the intrinsic G proteins, blocking signal transduction in both *in vitro* and *in vivo* cancer models (27). We validated the efficacy of the CXCR1/2-targeted pepducin, x1/2pal-i3, to attenuate CXCL-mediated tumourigenicity in *PTEN*-modulated prostate cancer cells, using an appropriate non-targeting peptide as the relevant control.

**Figure 5.**
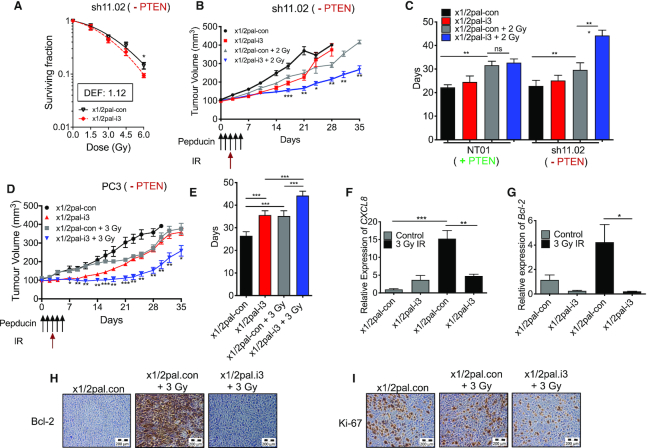
Inhibition of CXCR1/CXCR2 signalling delays tumour growth and potentiates RT-mediated growth delay of PTEN-null prostate cancer models. (**A**) Clonogenic survival curves showing DU145-sh11.02 survival fractions following treatment with increasing doses of IR. Cells were pre-treated 4 h prior to IR exposure with either control pepducin (x1/2pal-con) or CXCR1/2-targeted pepducin (x1/2pal-i3). (**B**) Tumour growth curves showing sh11.02 xenograft tumour volumes. Mice were randomized into four groups (*N* = 7 per group): x1/2pal-con, x1/2pal-i3, x1/2pal-con + 2 Gy and x1/2pal-ie + 2 Gy. Days in which pepducin (2 mg/kg) and IR treatments were performed are indicated. (**C**) Bar chart showing the mean time for PTEN-expressing NT01 and PTEN-deficient sh11.02 tumours to quadruple in size following treatment with the interventions modelled in (B). (**D**) Tumour growth curves showing PC3 xenograft tumour volumes. Mice were randomized into four groups (*N* = 7 per group): x1/2pal-con, x1/2pal-i3, x1/2pal-con + 3 Gy and x1/2pal-ie + 3 Gy. Days in which pepducin (2 mg/kg) and IR treatments were performed are indicated. (**E**) Bar chart showing the mean time for PC3 tumours to quadruple in size following treatment with the interventions modelled in (D). (**F**, **G**) Bar charts showing gene expression of *CXCL8* and *BCL2* following treatment of PC3 xenografts with the interventions modelled in (D). Tumours were harvested on study day 5 before extracting RNA for qRT-PCR analysis. Both graphs show mice treated with pepducin but no IR (grey bars) or a combination of pepducin and IR (black bars). (**H**, **I**) Images depicting Bcl-2 and Ki-67 IHC analysis of PC3 xenografts harvested on study day 5. Scale bars indicate 200 μm. Data information: all data presented are in the format of mean ± SE. For (A), statistically significant differences for individual dose points were determined using *t*-tests. For tumour growth analysis, statistically significant differences between radiation alone and combination treatment on specific study days were determined using *t*-tests (*P < 0.05; **P < 0.01; ***P < 0.001).

We initially validated the ‘pepducin’ approach *in vitro*; as expected, using our DU145-NT01 and sh11.02 models, we observed that the addition of x1/2pal-i3 only increased radiation sensitivity *in vitro* in the PTEN-deficient context (Figure 5A; Supplementary Figure S3C). Experiments were extended *in vivo* following implantation of sh11.02 cells in the flank of SCID mice. Tumours (100 mm^3^) were subjected to daily injections of either the x1/2pal-i3 targeting peptide or the control peptide (x1/2pal-con) for five consecutive days and a single 2 Gy radiation exposure on day 2. On day 21, mice treated with the control pepducin (x1/2pal-con) displayed a mean tumour volume of 369.6 ± 22.3 mm^3^. Treatment with x1/2pal-i3 alone resulted in a tumour growth delay after 21 days (226.1 ± 16.0 mm^3^), which was comparable to mice treated with 2 Gy IR (249.9 ± 15.9 mm^3^). However, the combined treatment consisting of 2 Gy IR and x1/2pal-i3 was most efficacious, resulting in a mean tumour volume of 166.6 ± 10.9 mm^3^ (Figure 5B). This combined therapy extended the median quadrupling time by 52% compared to tumours treated with IR tumours alone (*P* < 0.001; Figure 5C). Treatment of PTEN-expressing NT01 tumours with x1/2pal-i3 offered no additive effect in combination with IR (Supplementary Figure S3D).

Further experiments were conducted in a second tumour model of *PTEN*-null PC3. As anticipated, administration of the x1/2pal-i3 pepducin increased sensitivity to IR and repressed CXCL8-induced Bcl-2 expression *in vitro*, while use of a non-targeting pepducin (x1/2pal-con) had no effect (Supplementary Figure S3A and B). Once again, we observed a profound effect of the CXCR1/2-targeting pepducin upon the growth of *PTEN*-null PC3 xenograft tumours. Relative to tumours treated with the control pepducin (x1/2pal-con; mean tumour volume of 303.0 ± 27.8 mm^3^ after 21 days), treatment with either IR (3 Gy) or the receptor-targeted x1/2pal-i3 pepducin produced a similar tumour growth delay with 21-day mean tumour volumes of 198.7 ± 21.7 and 186.8 ± 6.7 mm^3^, respectively. However, the combined use of radiation and administration of the x1/2pal-i3 pepducin attenuated tumour growth to a mean volume of 111.8 ± 11.1 mm^3^ after 21 days (Figure 5D). Experiments were extended post 21 days and the tumour quadrupling time for each cohort was calculated (Figure 5E). There was no difference in tumour growth between the untreated control group (26.5 ± 1.9 days) and those treated with the non-targeting x1/2pal-con peptide (26 ± 1.3 days). Relative to x1/2pal-con-treated tumours, treatment with the CXCR1/2-targeting x1/2pal-i3 pepducin attenuated tumour growth (34.7 ± 0.91 days; *P* = 0.0003), producing a comparable growth delay to radiation (3 Gy; 35.7 ± 2.008 days). Combined treatment of 3 Gy plus x1/2pal-i3 produced a mean tumour growth delay of 43 ± 1.23 days, extending the time to reach the experimental endpoint by 20.4% over radiation alone. No acute toxicity was observed between the treatment groups as determined by changes in body weight in either the DU145 or PC3 models (Supplementary Figure S4A–C).

Pharmacodynamic markers were assessed in PC3 xenografted tumours harvested from additional mice within each experimental cohort at day 5 (48 h post-IR). As predicted by our prior *in vitro* data, qRT-PCR analysis of the tumours confirmed that exposure to IR exposure elevated gene expression of *CXCL8* and *BCL2*; this induction of gene expression was attenuated in the presence of the receptor-targeted x1/2pal-i3 pepducin (*P* = 0.005 and *P* = 0.031, respectively; Figure 5F and G). Furthermore, IHC performed on tumour sections confirmed that IR exposure increased Bcl-2 in these *PTEN*-null tumours, which was again reversed by x1/2pal-i3-mediated inhibition of CXCR1/2 signalling (Figure 5H). Moreover, inhibition of CXCR1/2 signalling impaired the proliferative capacity of irradiated PC3 tumours as shown by reduction in the Ki-67 positive cell population (Figure 5I).

### The CXCR2 antagonist AZD5069 combined with radiation slows tumour growth in a *PTEN*-null xenograft model

**Figure 6.**
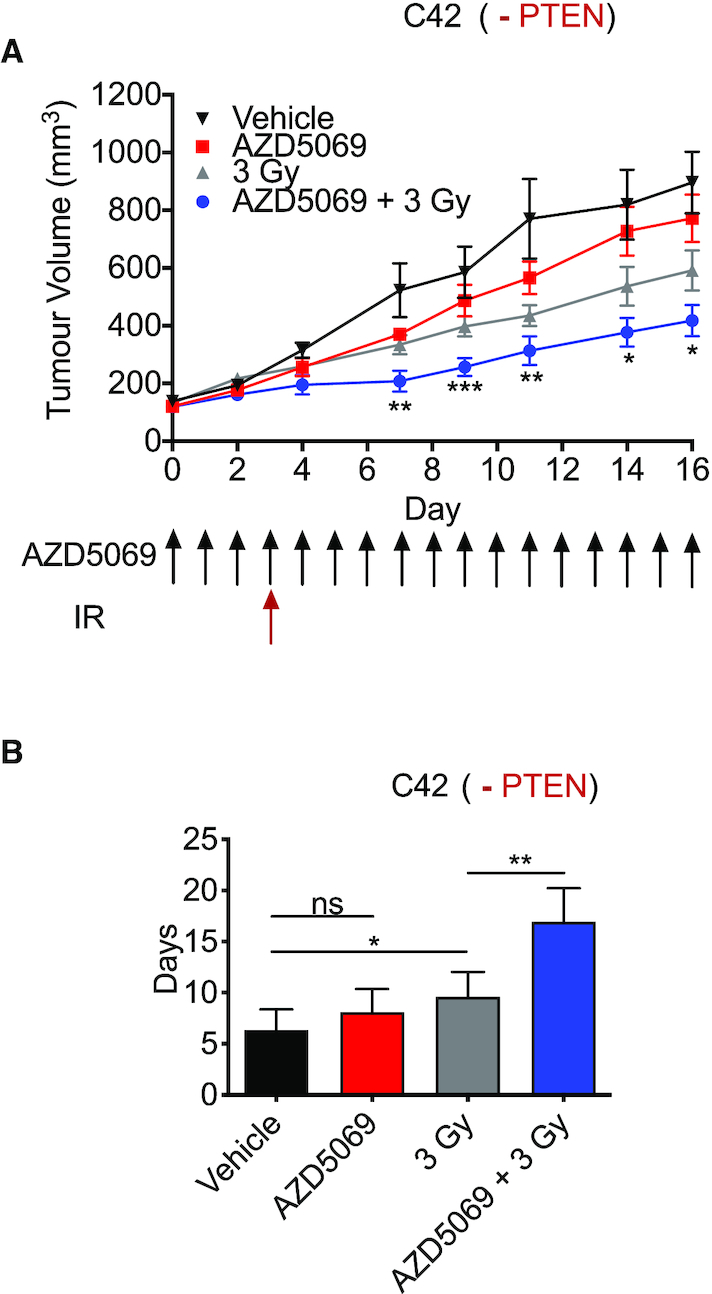
AZD5069-mediated CXCR2 inhibition increases C4-2 tumour radiosensitivity. (**A**) Tumour growth curves showing C4-2 xenograft tumour volumes. Mice were randomized into four groups (*N* = 8 per group): vehicle control, AZD5069, 3 Gy IR and AZD5069 + 3 Gy IR. (**B**) Bar chart showing the mean time for C4-2 tumours to quadruple in size following treatment with the interventions modelled in (A). Data information: all data presented are in the format of mean ± SE. For tumour growth analysis, statistically significant differences between radiation alone and combination treatment on specific study days were determined using *t*-tests (*P < 0.05; **P < 0.01; ***P < 0.001).

To further validate our observations using a molecular approach to abrogate CXCR2 signalling, we sought to use a pharmacological approach. AZD5069 is a selective, small-molecule CXCR2 receptor antagonist that has shown tolerability in respiratory medicine conditions and is currently undergoing clinical evaluation in solid tumours, including advanced metastatic castration-resistant prostate cancer (NCT03177187; ‘ACE’ Trial) (28). Accordingly, we used AZD5069 to determine whether a CXCR2-selective antagonist would phenocopy the impacts observed following administration of the receptor targeting pepducin. C4-2 tumours were established in SCID mice and treatment with either vehicle or AZD5069 started when tumours reached a volume of 100 mm^3^. Radiation (3 Gy) was delivered on day 3 of this treatment regime. Combined therapy resulted in a tumour growth delay compared to either AZD5069 alone or 3 Gy IR alone (Figure 6A). Tumour quadrupling times were calculated to determine the benefit of combined therapy. Mice treated with vehicle control had a tumour quadrupling time of 6.33 ± 2.04 days compared to 8.07 ± 2.29 days in mice treated with AZD5069 (ns; *P* = 0.181). Treatment with a single 3 Gy of IR resulted in a quadrupling time of 9.083 ± 2.94 days. However, the greatest response was seen in mice exposed to combined therapy (16.92 ± 3.31 days; *P* = 0.0014), extending the time to reach experimental endpoint by 86.31% (Figure 6B). Importantly, no acute toxicity was observed between the treatment groups as determined by changes in body weight over the course of the experiment (Supplementary Figure S4D).

## DISCUSSION

The use of radiation (external beam RT in locally advanced prostate cancer, stereotactic RT in the treatment of oligometastatic prostate cancer or radionuclides for resolution of bone disseminated castration-resistant prostate cancer) is a major treatment modality across the clinical landscape of prostate cancer. Technological advancements in the delivery and concentration of radioactivity to malignant zones of interest and use of devices to reduce radiation exposure to neighbouring tissues have greatly improved the response and tolerability of RT. However, over one-third of patients with organ-confined disease experience distant relapse, while radionuclides can extend survival but not overcome the incurable aspect of metastatic disease (29,30). To further improve the efficacy of RT, it is important to understand the biological mediators of RT resistance as foundational knowledge to characterize potential drug–RT combination regimens.

*PTEN* status has prognostic value in identifying patients at high risk of relapse post-RT (13). However, biological drivers underpinning PTEN-associated relapse are yet to be characterized or evaluated clinically. We have previously shown that *PTEN* loss selectively potentiates CXCL8 signalling in pre-clinical human and genetically engineered murine models of prostate cancer (21). The association of PTEN status with elevated chemokine signalling was confirmed by analysis of the MSKCC, TCGA and FASTMAN transcriptomic databases, which identified the presence of a PTEN^LOW^/CXCR1/2^HIGH^ cluster, which correlated with increased rates of BCR following either radical prostatectomy (MSKCC and TCGA) or radical RT with curative intent (FASTMAN). Moreover, we were able to establish the relationship of this PTEN^LOW^/CXCR1/2^HIGH^ cluster with distant metastasis in the FASTMAN cohort.

Several published studies have helped us to understand mechanisms by which *PTEN*-deficient cells escape radiation-induced cell death. These studies have mainly focused on the role of *PTEN* in the DNA damage response. McCabe *et al.* confirmed efficacy when combining RT with an ATM inhibitor (15), while other groups have debated the relationship between *PTEN* and *Rad51* (31). In the present study, we sought to examine whether *PTEN*-mediated inflammatory signalling could potentiate the radiation response in prostate cell models. Our data confirm that exposure of PTEN-deficient but not PTEN-expressing cells to clinically relevant doses of IR increases the expression of CXCL8, a chemokine that induces the activation of CXCR1 and CXCR2 receptors. Moreover, expression of both receptors was shown to increase in prostate cancer cell lines, albeit independent of their PTEN status.

Subsequent cell colony assays confirmed that the promotion of increased chemokine signalling was coupled to an adverse response of irradiated PTEN-deficient prostate cancer cells, and consequently, that the inhibition of this chemokine signalling pathway markedly increased the sensitivity of three distinct models of prostate carcinoma to IR. The use of isogenic cells with siRNA-, shRNA- or tetracycline-induced differential expression of PTEN confirmed that the abrogation of chemokine signalling only conferred radiosensitization in the context of *PTEN* deficiency. Furthermore, a more pronounced effect of inhibiting chemokine signalling was observed at higher doses of IR. This is consistent with the clinical scenario that is rapidly shifting toward hypofractionated or stereotactic RT protocols and utilizes higher doses of IR (10).

Due to the well-established role of PTEN in the DNA damage response, we sought to determine whether elevated chemokine signalling could modulate DNA damage repair. Interestingly, inhibition of CXCR1/2 signalling *in vitro* did not modulate the magnitude of DNA foci formation or the rate of DNA repair in these cells. Recent research has confirmed that elevated CXCR2 expression can facilitate unfavourable prognosis and tumourigenesis via activation of the JAK2/STAT3 pathway (32). Our data suggest that radiation-induced CXCR1/2 signalling sustains the viability of *PTEN*-depleted cells using this exact mechanism. Inhibition of CXCR1/2 signalling prior to IR exposure in *PTEN*-null LNCaP and *PTEN*-depleted DU145 cells significantly induced apoptosis, characterized by Annexin V/PI flow cytometry analysis and the induction of caspase and PARP cleavage. Targeting CXCR1/2 prevented JAK2 and STAT3 phosphorylation, consistent with the observations of Wei *et al.* (32). These findings are in line with our prior studies confirming that inhibition of CXCR2 signalling potentiated oxaliplatin-induced apoptosis in PC3 cells (23).

Our *in vivo* experimentation in three distinct prostate tumour models provides further compelling evidence for the contribution of CXCR1/CXCR2 signalling in adversely affecting RT outcome. Administration of a dual CXCR1/2-targeted antagonistic peptide alone inhibited the growth of *PTEN*-deficient but not *PTEN*-expressing DU145 and PC3 xenograft tumours, consistent with our prior and current demonstration of CXCL8 functioning as a key survival factor in this genetic context (21). Interestingly, the CXCR1/2-targeted pepducin was equi-effective in enabling an antitumour response as a clinically relevant dose of radiation in both PTEN-depleted DU145 and PC3 tumours. However, of even greater significance, we observed that peptide-mediated inhibition extended the growth delay afforded by exposure of both *PTEN*-depleted DU145 and PC3 models to IR. These observations were further validated by experimentation in a third model, wherein the sensitivity of the LNCaP C4-2 to IR was increased by the administration of the small-molecule antagonist of the CXCR2 receptor, AZD5069. Therefore, we have shown consistent responses across three distinct PTEN-deficient prostate cancer models, using both molecular and pharmacological interventions.

The pronounced effect of targeting CXCR1 and CXCR2 signalling observed in our *in vivo* models is consistent with the multifactorial role of chemokines within the tumour microenvironment. The baseline and radiation-enhanced secretion of CXCL8 (and its orthologues CXCL1, CXCL2 and CXCL5) from PTEN-deficient prostate tumour epithelial cells exerts both autocrine and paracrine actions within the tumour microenvironment given the expression of CXCR1 and CXCR2 upon multiple cell types (33,34). First, our *in vitro* data confirm a radiation-induced potentiation of CXCR1 and CXCR2 signalling in PTEN-deficient cells, which increases anti-apoptotic gene and protein expression. Analysis of pharmacodynamic markers within harvested PTEN-deficient tumour samples similarly indicated IR to induce the expression of Bcl-2, a known mediator of RT resistance in a number of cancer models, but that this was attenuated by inhibition of CXCR1/CXCR2 signalling (35). Second, tumour-derived CXCL signalling is likely to exert paracrine activation on surrounding stromal fibroblasts and monocyte-derived immune cells (14). Recent studies have confirmed that inhibition of CXCR2 signalling can repress the activity of myeloid-derived suppressor cells and re-educate the differentiation of tumour-associated macrophages in genetically engineered mouse models (36). The broader significance of inhibiting CXCR signalling upon the constitution and activity of the tumour microenvironment following exposure to RT is worthy of future targeted and more comprehensive investigation.

In conclusion, we have characterized a distinct cluster of primary prostate cancer defined by *PTEN*^LOW^, CXCR1^HIGH^ and CXCR2^HIGH^ expression that associates with adverse downstream clinical outcome. We have provided further experimental evidence using established models of disease that this biology may be a functional driver of the impaired RT response observed in PTEN-deficient tumours. Combined, our *in vitro* and *in vivo* data demonstrate that subjecting *PTEN*^LOW^ cancer cells or tumours to clinically relevant doses of IR amplifies chemokine signalling, potentiating autocrine and paracrine signalling throughout the microenvironment that supports the survival of PTEN-deficient cells and facilitates the acquisition of a myeloid-enriched and potentially immunosuppressive microenvironment. Molecular-mediated and pharmacological-based inhibition of CXCR1/CXCR2 signalling sensitized each of the three PTEN-deficient tumour models to clinically relevant doses of IR. Consequently, we propose that targeting this chemokine signalling pathway may constitute a relevant therapeutic strategy to enhance the response of *PTEN*-deficient prostate carcinomas to radiation therapy. Given that functional impairment of *PTEN* is reported in up to 30% of all primary prostate cancers and that radical RT is a major treatment option for locally advanced high-risk disease, CXCR1/CXCR2-targeted therapeutics may have a significant impact across a large cohort of patients.

Furthermore, the observation that chemokine receptor inhibition may have greater impacts at even higher radiation doses suggests that this intervention may have even greater significance in respect of the use of stereotactic RT in resolution of oligometastatic disease, and potentially, where there is further enrichment in the prevalence of *PTEN* gene aberration. CXCR2 antagonist AZD5069 is currently undergoing clinical evaluation in a prostate cancer setting. Pending initial results relating to both toxicity and efficacy, we propose that further examination is warranted alongside RT.

## Supplementary Material

zcaa012_Supplemental_Files
