# Supplementary material for: Clinical and functional characterization of CXCR1/CXCR2 biology in the relapse and radiotherapy resistance of primary PTEN-deficient prostate carcinoma
Source: NAR Cancer. 2020 Jul 3;2(3):zcaa012. doi: 10.1093/narcan/zcaa012 (PMC7380483; doi:10.1093/narcan/zcaa012)
Supplement: zcaa012_Supplemental_Files [file zcaa012_supplemental_files.zip › Supplementary Figures Legends and Tables.docx]

**Supplementary Figure Legends**

**Supplementary Figure 1. Restoration of PTEN expression abrogates CXCR1/2-mediated radiosensitivity.**

**A-B** Clonogenic survival curves generated from PC3 and PC3+PTEN cells respectively. Cells were pre-treated with 25nM non-targeting siRNA (siNT) or CXCR1 and CXCR2 siRNA (siCXCR1/2) for 48H prior to IR treatment.

Data information: All data presented is a representation of N=3 independent experiments. All survival curves are fitted to a linear quadratic model and Dose Enhancement Factor (DEF) was calculated using the mean inactivation dose based on the area under the curve at a surviving fraction of 0.1. Statistically significant differences were determined using t-tests (*p*<0.05*; *p*<0.01**; *p*<0.001***).

**Supplementary Figure 2. Loss of PTEN expression impairs DNA damage repair but CXCR1/2 inhibition has no effect on the DNA damage response.**

**A** Immunofluorescence images showing 53BP1 foci formation (red) in DU145 NT01 and sh11.02 cells at several timepoints following treatment with 1 Gy IR. Nucleus staining (blue) is also shown via the use of DAPI.

**B** Bar chart showing the average number of foci per cell under control conditions in both NT01 and sh11.02 cells.

**C** Graph presenting the kinetic profile of double-strand break repair in *PTEN*-functional NT01 and *PTEN*-depleted sh11.02 DU145 cells over a 24H time-period following exposure to ionizing radiation (1 Gy).

**D-E** Bar charts showing average number of 53BP1 foci in NT01 and sh11.02 cells respectively under control conditions or 4H post IR treatment. Cells were pre-treated with non-targeting siRNA (black bars) or CXCR1/2 siRNA (grey bars) to determine the effect of CXCR1/2-inhibition on the DNA damage response.

Data information: All data presented is a representation of N=3 independent experiments. Statistically significant differences were determined using t-tests (*p*<0.05*; *p*<0.01**; *p*<0.001***). No significant difference is indicated using *ns*.

**Supplementary Figure 3. CXCR1/2-targeted pepducins in PTEN-null and PTEN-expressing prostate cancer cell lines.**

**A** Clonogenic survival curves showing PC3 survival fractions following treatment with increasing doses of IR. Cells were pre-treated 4H prior to IR exposure with either control pepducin (x1/2pal-con) or CXCR1/2-targeted pepducin (x1/2pal-i3).

**B** Bar chart showing *BCL2* gene expression under control conditions (grey bars) or following addition of 3nM recombinant human CXCL8 (black bars). Cells were pre-treated 4H prior to IR exposure with either control pepducin (x1/2pal-con) or CXCR1/2-targeted pepducin (x1/2pal-i3).

**C** Clonogenic survival curves showing DU145 NT01 survival fractions following treatment with increasing doses of IR. Cells were pre-treated 4H prior to IR exposure with either control pepducin (x1/2pal-con) or CXCR1/2-targeted pepducin (x1/2pal-i3).

**D** Tumor growth curves showing NT01 xenograft tumor volumes. Mice were randomized into four groups (N=7/group): x1/2pal-con; x1/2pal-i3; x1/2pal-con + 2 Gy; and x1/2pal-ie + 2 Gy. Days in which pepducin (2 mg/kg) and IR treatments were performed are indicated.

Data information: All data presented is in the format of mean ± SE. For (A) statistically significant differences for individual dose points were determined using t-tests. For tumor growth analysis statistically significant differences between radiation alone and combination treatment on specific study days were determined using t-tests (*p*<0.05*; *p*<0.01**; *p*<0.001***).

**Supplementary Figure 4. Body weight assessment following treatment with CXCR1/2-targeted agents alongside radiotherapy.**

**A-C** Graphs showing percentage body weight change relative to study day 0 in mice implanted with DU145 NT01, sh11.02 or PC3 cells respectively. Each graph shows the effect of the following treatments on body weight: x1/2pal-con; x1/2pal-i3; x1/2pal-con + 2 Gy IR; or x1/2pal-i3 + 2 Gy IR.

**D** Graph showing percentage body weight change in mice implanted with C4-2 cells following treatment with vehicle control, AZD5069, 3 Gy IR or AZD5069 + 3 Gy IR (Combination).

Data information: No significant difference was observed in any model as determined by ANOVA.

**Supplementary Figure 5. *PTEN^LOW^*, *CXCR1^HIGH^* and *CXCR2^HIGH^* tumors are associated with poor prognosis in TCGA prostate cancer dataset.**

**A** Cluster analysis of *PTEN*, *CXCR1* and *CXCR2* in the TCGA dataset.

**B** Kaplan-Meier survival curves in the TCGA dataset in relation to sample clustering by three genes (*PTEN*, *CXCR1* and *CXCR2*).

Data information: Significant differences were determined by log-rank test. Abbreviations: BCR, biochemical recurrence; HR, hazard ratio.

**Supplementary Table 1. Western blot antibody information.**

| Antibody | Supplier | Cat no. | Dilution |
| --- | --- | --- | --- |
| Akt | Cell Signaling | 9272 | 1:1000 |
| pAkt (Ser473) | Cell Signaling | 9271 | 1:1000 |
| β-Actin | Cell Signaling | 3700 | 1:5000 |
| Bcl-2 | Santa Cruz | sc-7382 | 1:2500 |
| Cleaved Caspase 9 | Cell Signaling | 7237 | 1:1000 |
| CXCL8 | R&D Systems | MAB208 | 1:750 |
| CXCR1 | R&D Systems | MAB330 | 1:750 |
| CXCR2 | R&D Systems | MAB331 | 1:750 |
| JAK2 | Abcam | ab108596 | 1:1000 |
| pJAK2 (Tyr1007/8) | Abcam | ab32101 | 1:1000 |
| PARP | Cell Signaling | 9542 | 1:1000 |
| PTEN | Cell Signaling | 9559 | 1:1000 |
| STAT3 | Abcam | ab68153 | 1:1000 |
| pSTAT3 (Tyr705) | Abcam | ab76315 | 1:1000 |

**Supplementary Table 2. Cell doubling times (± SEM) for *PTEN*-functional and ablated DU145 cells.**

| Cell line | 0 Gy NT | 0 Gy siCXCR1/2 | 3 Gy + siNT | 3 Gy siCXCR1/2 |
| --- | --- | --- | --- | --- |
| DU145 NT01 (+PTEN) | 2.163 ± 0.022 | 2.118 ± 0.021 (♯ ***) | 2.453 ± 0.025 | 4.627 ± 0.021 (♯ ***) (♮ ***) |
| DU145 sh11.02  (-PTEN) | 2.059 ± 0.041 | 2.148 ± 0.022  (α ***) | 2.238 ± 0.024 (♭***) | 6.853 ± 0.033 (α ***) (♭***) (♮ ***) |

Cell growth curves were established and monitored over a 10-day period. Populations were exposed to either sham-radiation (0 Gy) or radiation (3 Gy), in the absence (siNT) or presence (siCXCR1/2) of a CXCR1/CXCR2-targeted siRNA strategy.

Raw data was fit to an exponential growth equation (Prism 6 software) from which the mean doubling time for each cell population was calculated. Statistically-significant differences in cell doubling were determined using a two-way ANOVA.

♯ represents a statistically-significant difference afforded by the provision of radiation to CXCR1/2-ablated, PTEN-expressing NT01-transfected cells.

α represents a statistically-significant difference afforded by the provision of radiation to CXCR1/2-ablated, PTEN-ablated sh11.02 cells.

♭represents a statistically-significant difference afforded by CXCR1/2-ablation in irradiated (3 Gy) PTEN-ablated sh11.02 cells (*p*<0.0001).

♮ represents a statistically-significant difference afforded by combined radiation and CXCR1/2-ablation in PTEN-deficient cells (*p*<0.0001).
