# Supplementary figures and images for: Clinical and functional characterization of CXCR1/CXCR2 biology in the relapse and radiotherapy resistance of primary PTEN-deficient prostate carcinoma
Source: NAR Cancer. 2020 Jul 3;2(3):zcaa012. doi: 10.1093/narcan/zcaa012 (PMC7380483; doi:10.1093/narcan/zcaa012)

Supplementary Figure 1

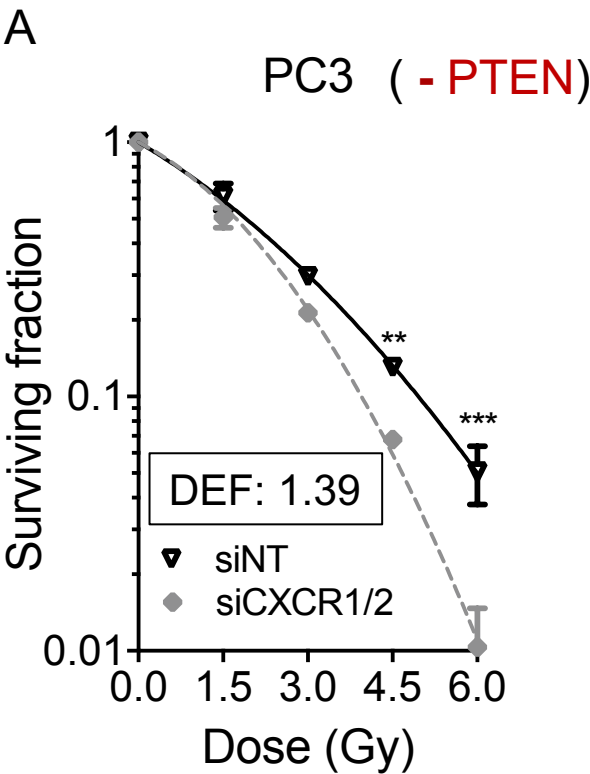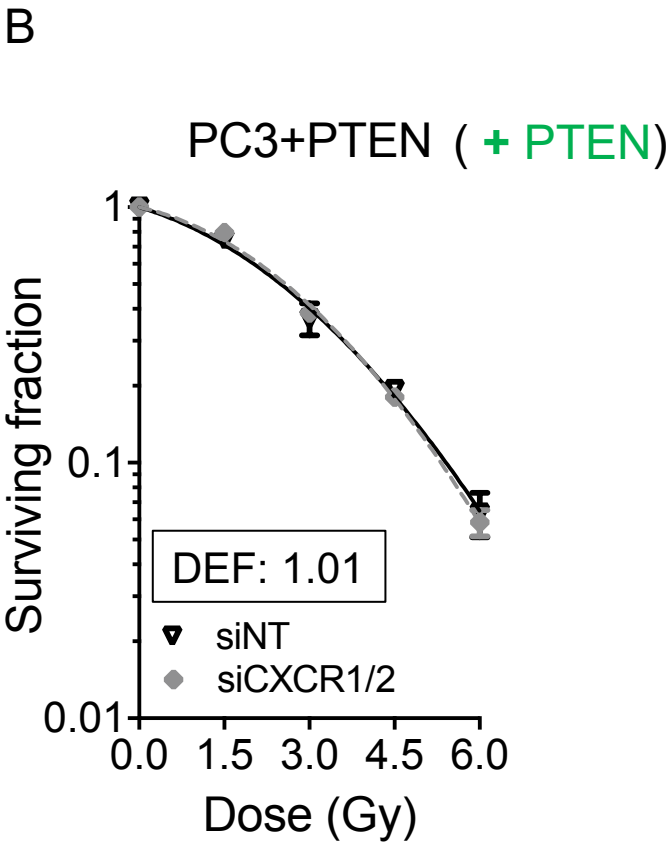

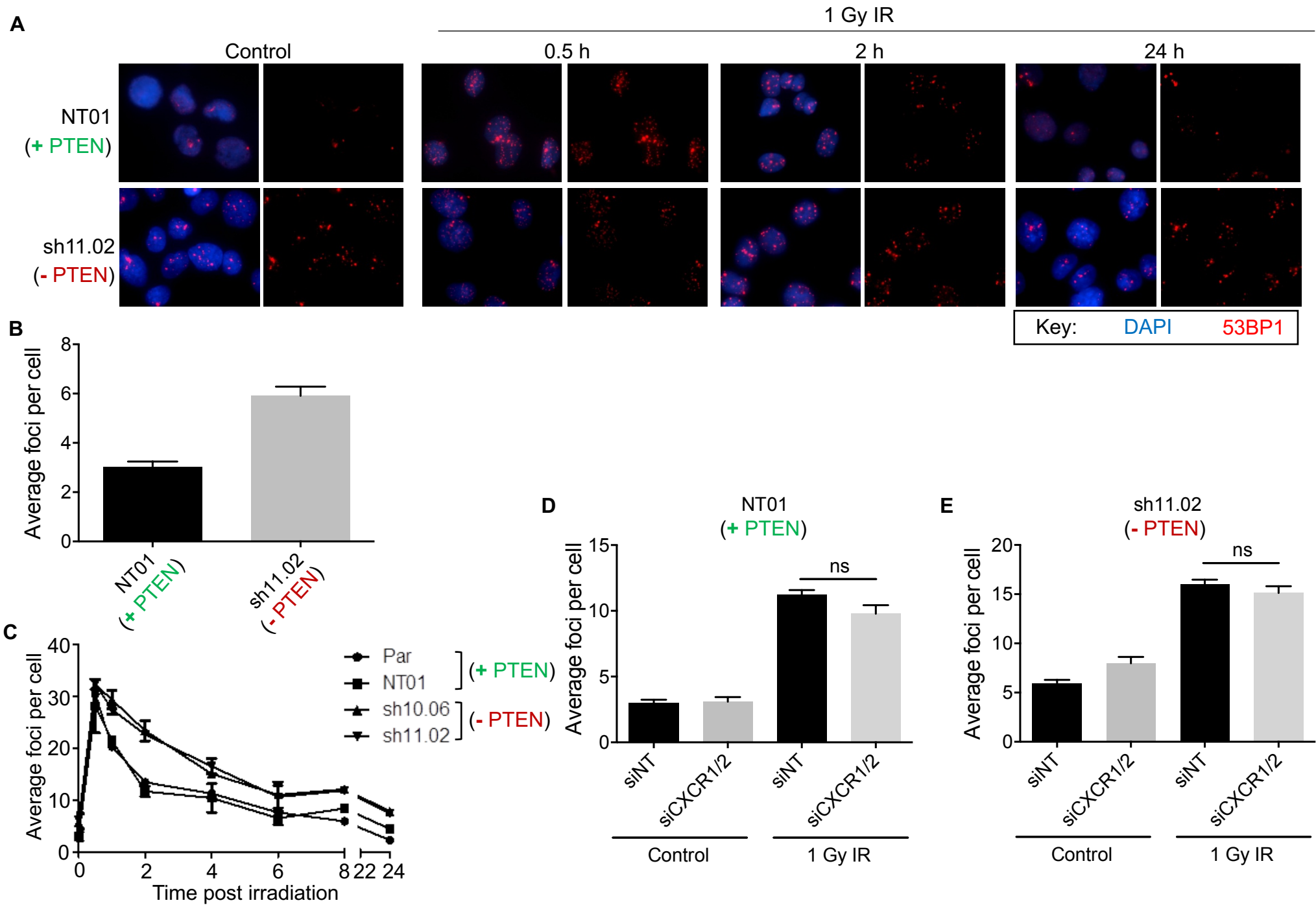

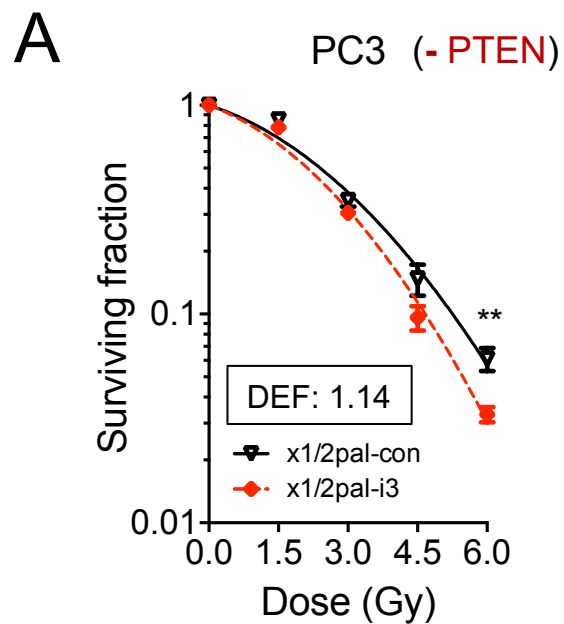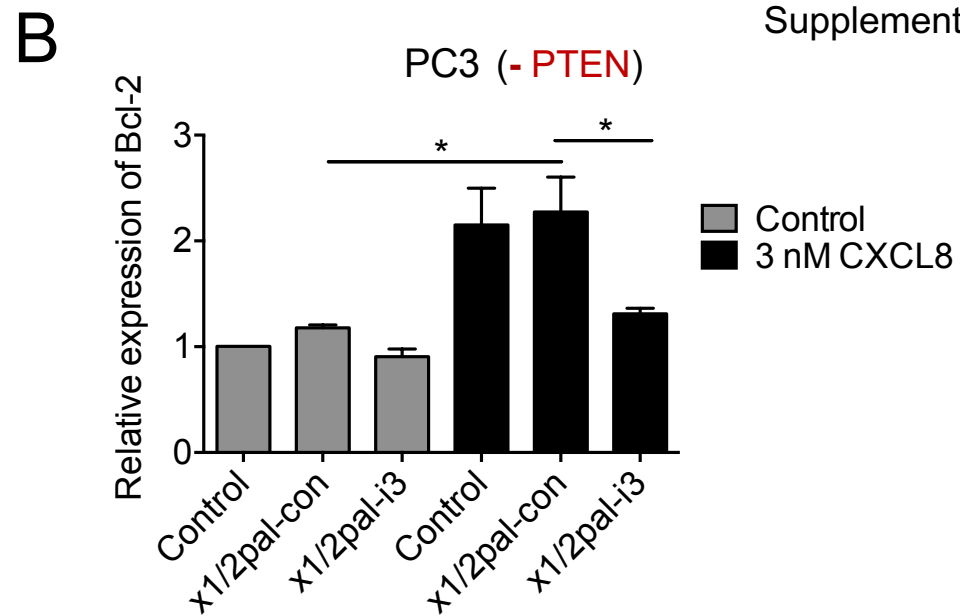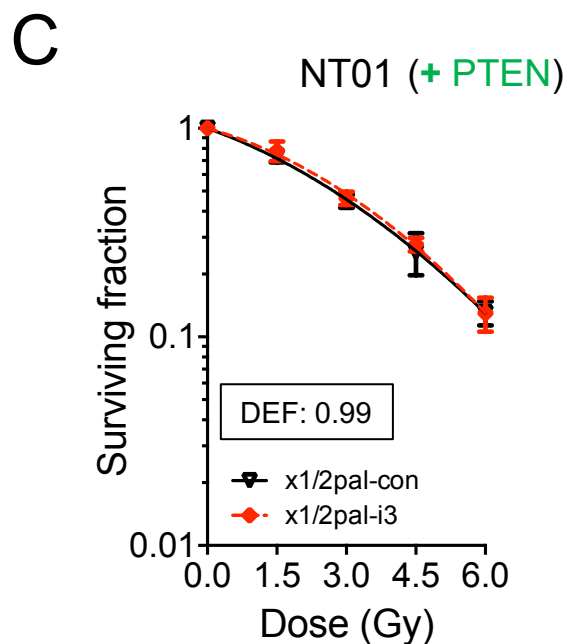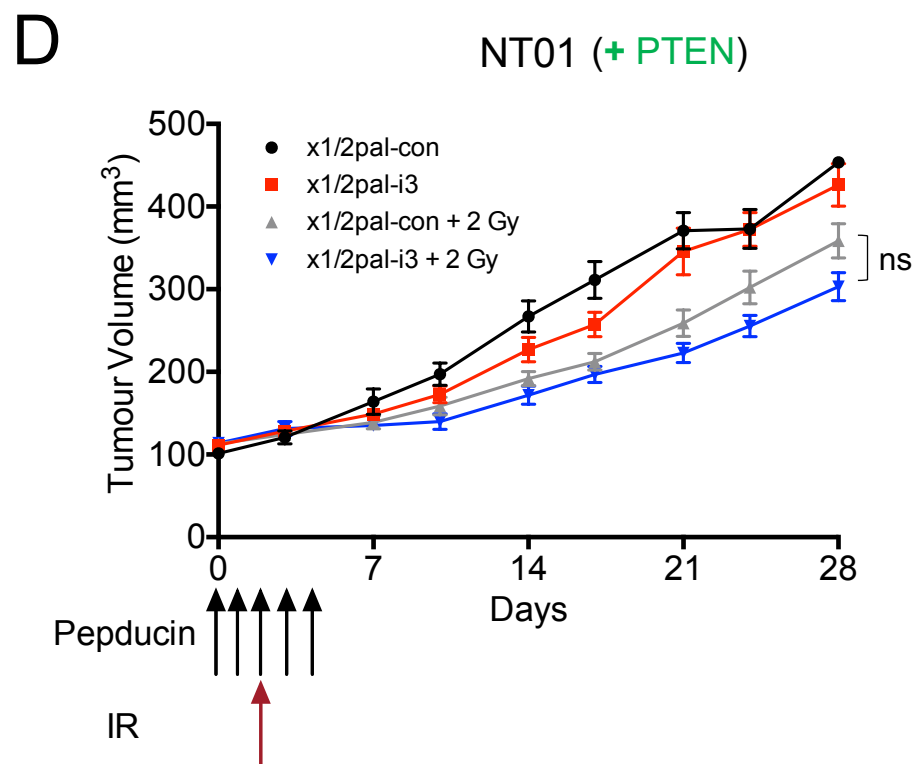

# Supplementary Figure 4

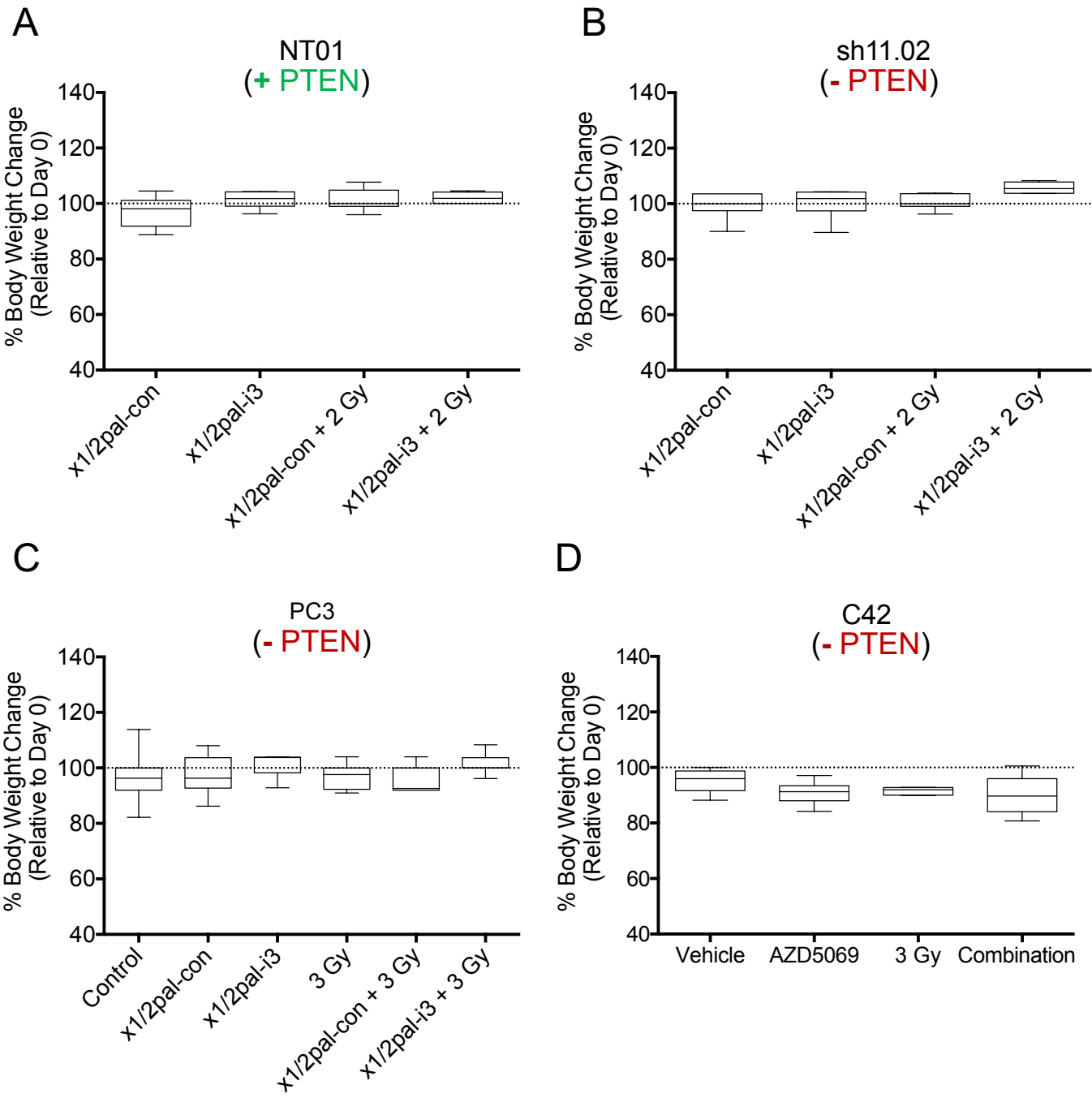

Supplementary Figure 5

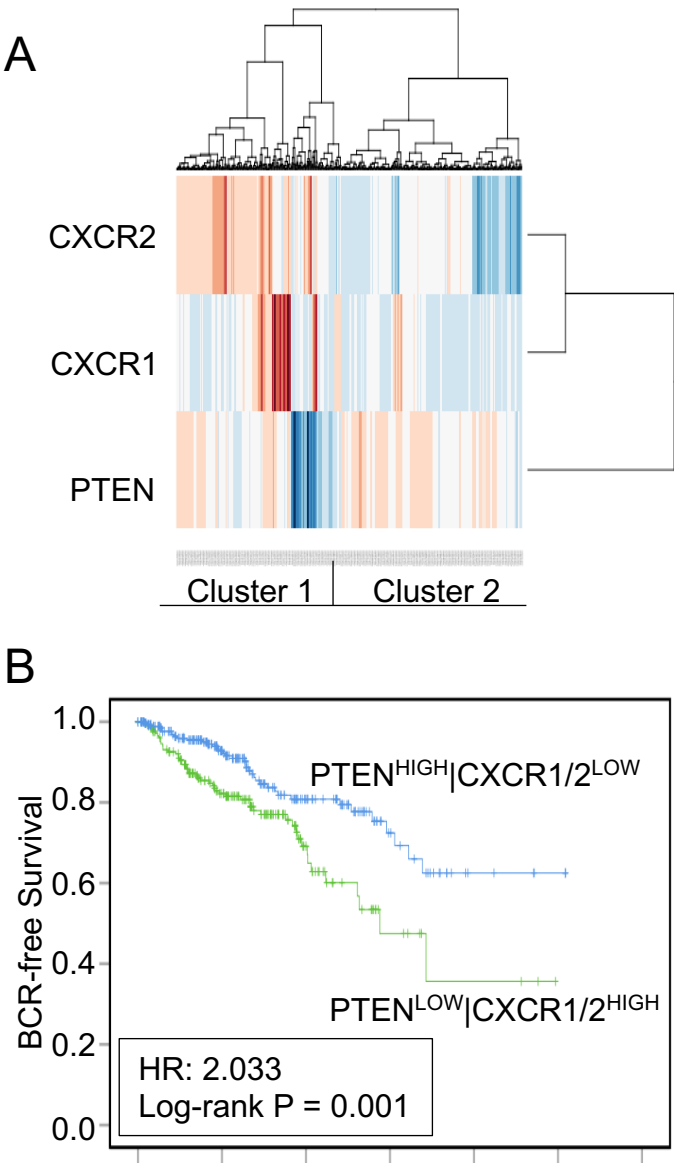

Supplement: zcaa012_Supplemental_Files [file zcaa012_supplemental_files.zip › Suppl Fig ALL_Revised_Merged.pdf]
